# Synthesis, crystal structure, Hirshfeld surface analysis, density function theory calculations and photophysical properties of methyl 4′-[(4-bromobenzo­yl)­oxy]biphenyl-4-carboxyl­ate: a compound with bromine⋯oxygen contacts

**DOI:** 10.1107/S2056989025001604

**Published:** 2025-02-28

**Authors:** Hanumaiah Anilkumar, Selvaraj Selvanandan, Metikurke Amruthesh Omkariah, Mahadevaiah Harish Kumar, Hosapalya Thimmaiah Srinivasa, Bandrehalli Siddagangaiah Palakshamurthy

**Affiliations:** a Department of Physics, Government First Grade College, Chikkballapur, Karnataka-562101, India; b Department of Physics, ACS College of Engineering, Bangalore, Karnataka-560074, India; c Department of Physics, Government First Grade College, Kadur, Karnataka-577548, India; d Department of Physics, Government Engineering College, Ramanagara, 562159, Karnataka, India; e Raman Research Institute, C V Raman Avenue Sadashivanagar Bangalore Karnataka India; f Department of PG Studies and Research in Physics Albert Einstein Block UCS Tumkur University, Tumkur Karnataka-572103 India; Vienna University of Technology, Austria

**Keywords:** crystal structure, physical properties, Hirshfeld surface, density function theory, biphenyl-4-carboxyl­ate and solvatochromic

## Abstract

In the mol­ecular title compound, C_21_H_15_BrO_4_, the dihedral angles between the aromatic bromo-benzene ring and the immediate neighbors (first and second aromatic ring of the biphenyl moiety) are 56.57 (2) and 50.91 (4)°. The dihedral angle between the aromatic rings of the biphenyl fragment is 5.78 (4)°. The torsion angles across the ester groups associated with bromo-benzene and methyl moieties are 178.0 (1) and 176.86 (2)°, respectively, revealing an *anti*-periplanar conformation in both cases. In the crystal, the packing of the mol­ecules is stabilized by Br⋯O contacts running infinitely along [001]. In addition, the crystal packing is consolidated by various C—H⋯π inter­actions. Hirshfeld surface analysis revealed that the most important contributions to the crystal packing arise from H⋯H (27.1%), C⋯H/H⋯C (39.3%), O⋯H/H⋯O (15.4%) and Br⋯H/H⋯Br (10.6%) contacts. The net inter­action energies for the title compound were computed as *E*_ele_ = −41.9 kJ mol^−1^, *E*_pol_ = −11 kJ mol^−1^, *E*_dis_ = −209.7 kJ mol^−1^ and *E*_rep_ = 108.9 kJ mol^−1^, with a total inter­action energy *E*_tot_ of −167.9 kJ mol^−1^. The ground-state dipole moment (μ_g_) is calculated as 1.2936 debye and the energy gap between HOMO and LUMO orbitals is 4.5203 eV as calculated with density functional theory using the B3LYP/6–31 G level basis set. The electronic absorption and fluorescence spectra of the compound were recorded and studied in different solvents by varying polarity. These results were used to elucidate the solvatochromic properties, and spectral deviations were studied by the linear solvation energy relationship. Lippert, Bakhshiev, and Bilot–Kawski–Chamma–Viallet equations were used to estimate the ground and excited-state dipole moments (μ_e_). The excited dipole moment is found to be higher than the ground state dipole moment, which indicates that π-electrons are more distributed in polar excited mol­ecules.

## Abstract 2

Br⋯O contacts between neighboring mol­ecules are present in the crystal of the title compound. Its photophysical properties were estimated by solvatochromic method.

## 1. Chemical context

Mol­ecules derived from biphenyl are found to exhibit liquid-crystal properties because of their linearity, high symmetry and thermal stability (Ranganathan & Ramesh, 2006 ▸; Imai *et al.*, 2001 ▸). The thermotropic liquid crystalline phases containing biphenyl moieties have an ability to form ordered structures so that they have been widely studied in recent decades (Bagheri *et al.*, 2004 ▸). The methyl­ene entity that is directly attached to biphenyl mesogens undergoes self-polycondensation, revealing smectic (Sm) A and B phases (Nakata & Watanabe, 1994 ▸). The absence of alkyl chains/highly polar groups at the ends in the mol­ecular structures of liquid crystals induces non-liquid crystal properties (Harish Kumar *et al.*, 2024*b* ▸). Rigid cores such as cyclic π- or heterocyclic π-systems are responsible for electro-optical phenomena. For example, biphenyl-4-carboxyl­ate derivatives are found to exhibit liquid-crystal properties, which play an important role in electro-optical phenomena caused by weak electric fields (Mikulko *et al.*, 2006 ▸). It is well known that mol­ecules derived from conjugated biphenyl-4-carboxyl­ate exhibit optical non-linear­ity, especially when they have donor and acceptor substituents at each end of the mol­ecular systems. This originates from an efficient intra­molecular charge transfer through a highly polarizable π-electron system. The electro-optical response depends on the structure, sample thickness, birefringence, light absorption, scattering and other factors. Therefore, the results of electro-optical measurements are in most cases arbitrary and, consequently, the absolute values of neither linear nor higher order electro-optical coefficients can be determined (Dardas *et al.*, 2009 ▸). However, the knowledge of linear and non-linear electro-optical coefficients are required to study the material response to an applied electric field. In this scenario it is necessary to look at the dipole moment, which has an influence on the refractive index or polarization changes at high electric field. Keeping this in mind, we made an attempt to synthesize corresponding phases and report here on the crystal structure analysis, solvatochromism response and dipole moment of the title compound, C_21_H_15_BrO_4_, (I).
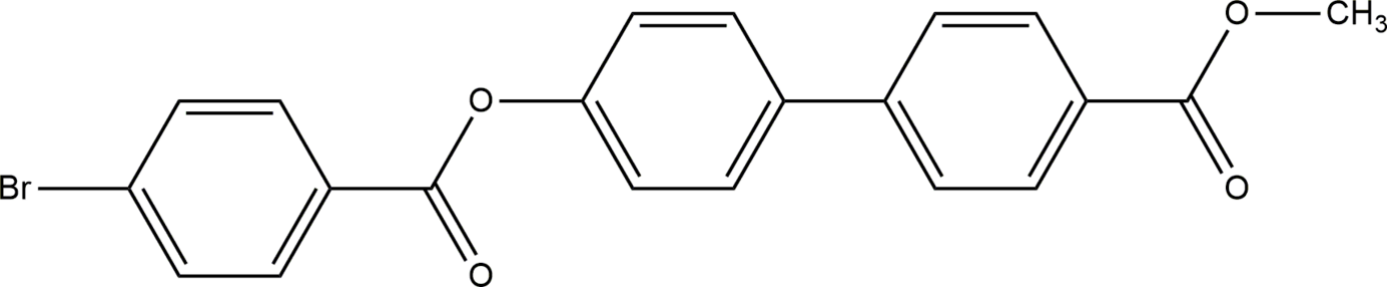


## 2. Structural commentary

**Figure 1:**
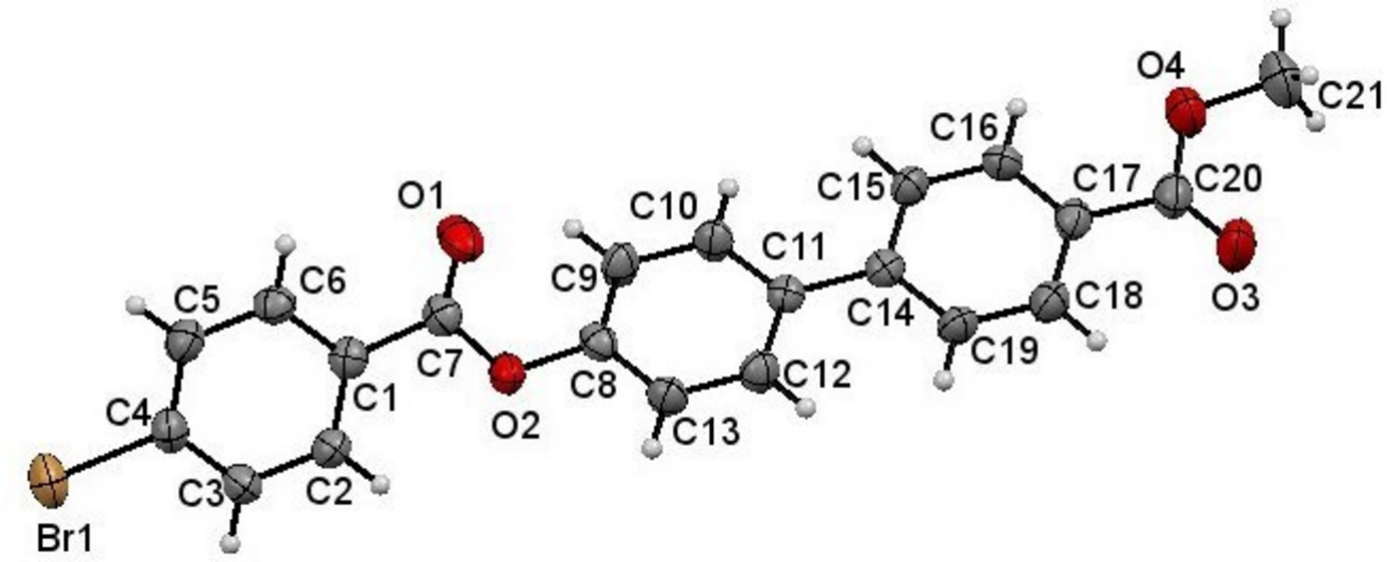
The mol­ecular structure of (I), showing displacement ellipsoids at the 50% probability level.

The mol­ecular structure of (I) is shown in Fig. 1 ▸. The dihedral angles between the bromo­benzene and the aromatic rings (C8–C13) and (C14–C19) of the biphenyl moiety are 56.57 (2) and 50.91 (4)°, respectively, whereas the dihedral angle between the two aromatic rings of the biphenyl moiety is 5.78 (4)°. The torsion angles involving the biphenyl moiety and the attached ester groups are 178.0 (3)° (C1—C7—O2—C8) and 176.9 (4)° (C17—C20—O4—C21), respectively, making the conformation *anti-*periplanar in both cases.

## 3. Supra­molecular features

**Figure 2:**
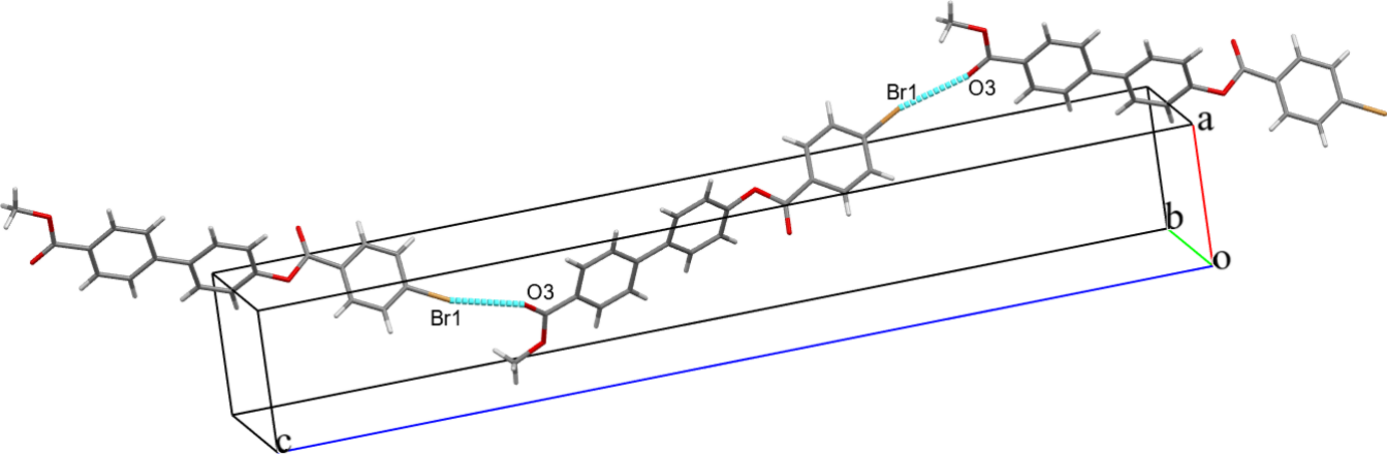
The mol­ecular packing of (I). Dashed lines indicate Br⋯O inter­actions.

**Figure 3:**
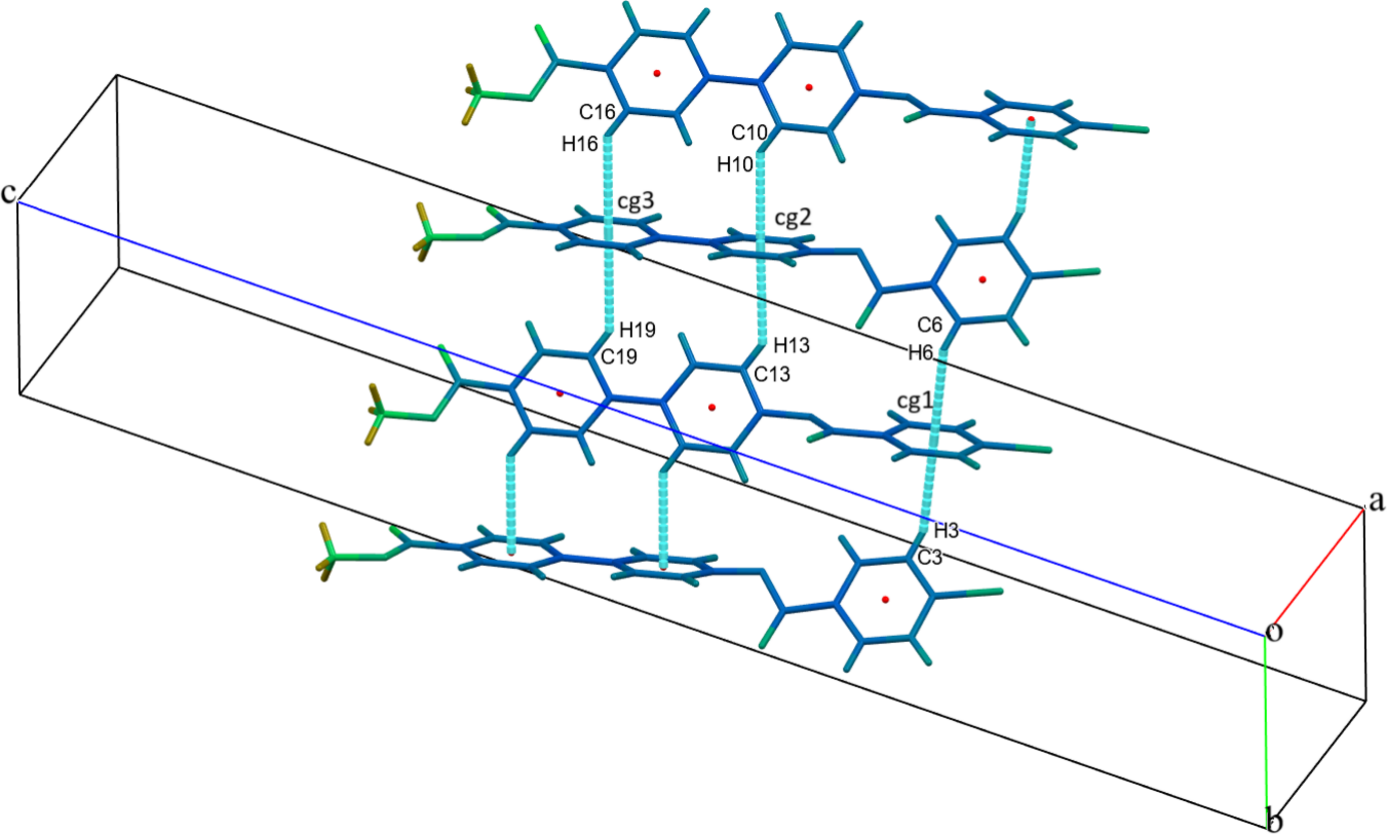
The mol­ecular packing of (I). Dashed lines indicate C—H⋯*Cg* inter­actions.

The crystal packing is stabilized by a halogen–oxygen (Br⋯O) inter­action C—Br1⋯O3—C with a Br⋯O distance of 3.105 (2) Å, forming infinite chains running parallel to [001] (Fig. 2 ▸). The packing is further consolidated by six C—H⋯π inter­actions between aromatic CH groups and the centroids (*Cg*) of adjacent aromatic rings (Table 1 ▸), as illustrated in Fig. 3 ▸.

## 4. Database survey

A search of the Cambridge Structural Database (CSD, version 5.42, update of November 2020; Groom *et al.*, 2016 ▸) for mol­ecules containing the biphenyl carboxyl­ate fragment resulted in more than thirty matches, with mol­ecular features similar to (I) for compounds with refcodes DEZYUF (Ardeleanu *et al.*, 2018 ▸), DOJLIB (Lin *et al.*, 2024 ▸), FIRYEN (Royal & Baudoin, 2019 ▸) and VUCFEI (Harish Kumar *et al.*, 2024*a* ▸). In these compounds, the dihedral angle between the aromatic rings of the biphenyl moieties are in the range of 33.07 (3) to 38.14 (5)°. They have simple substituent groups at one end of the biphenyl moiety. Compounds with refcodes COFMET (Cai *et al.*, 2024 ▸), ASUJAB (Lustig *et al.*, 2016 ▸), CUXCAC (Das *et al.*, 2021 ▸) all have substituents in the biphenyl fragments, with dihedral angles between the aromatic rings of the biphenyl moiety in the range 44.04 (3) to 51.06 (2)°. It is quite characteristic that the dihedral angle between unsubstituted biphenyl rings are around 30 to 45° due to steric hindrance between *ortho*-hydrogen atoms of each ring. The small value of 5.78 (4)° for the dihedral angle found in the title compound is due to the presence of bulky groups at each end of the mol­ecule and the inter­actions resulting from halogen⋯oxygen contacts at one end of the biphenyl moiety.

## 5. Hirshfeld surface analysis

**Figure 4:**
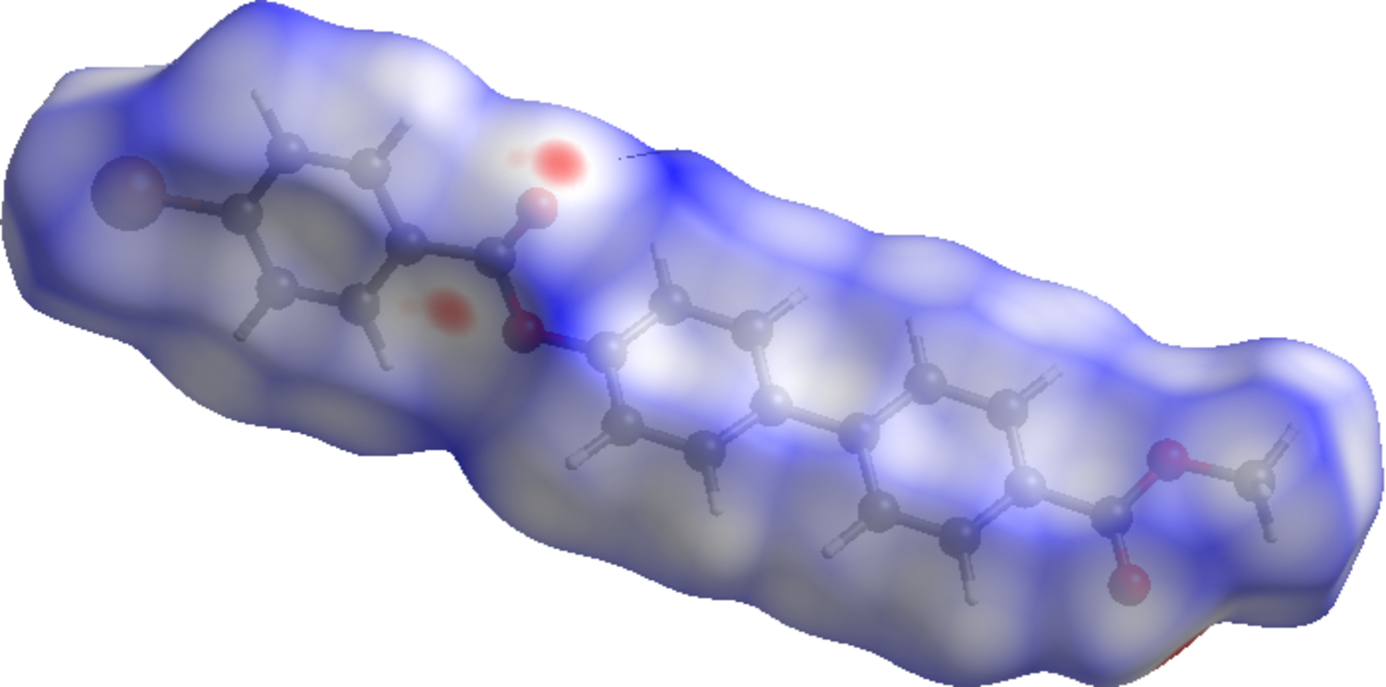
Hirshfeld surface of (I) mapped with *d*_norm_.

**Figure 5:**
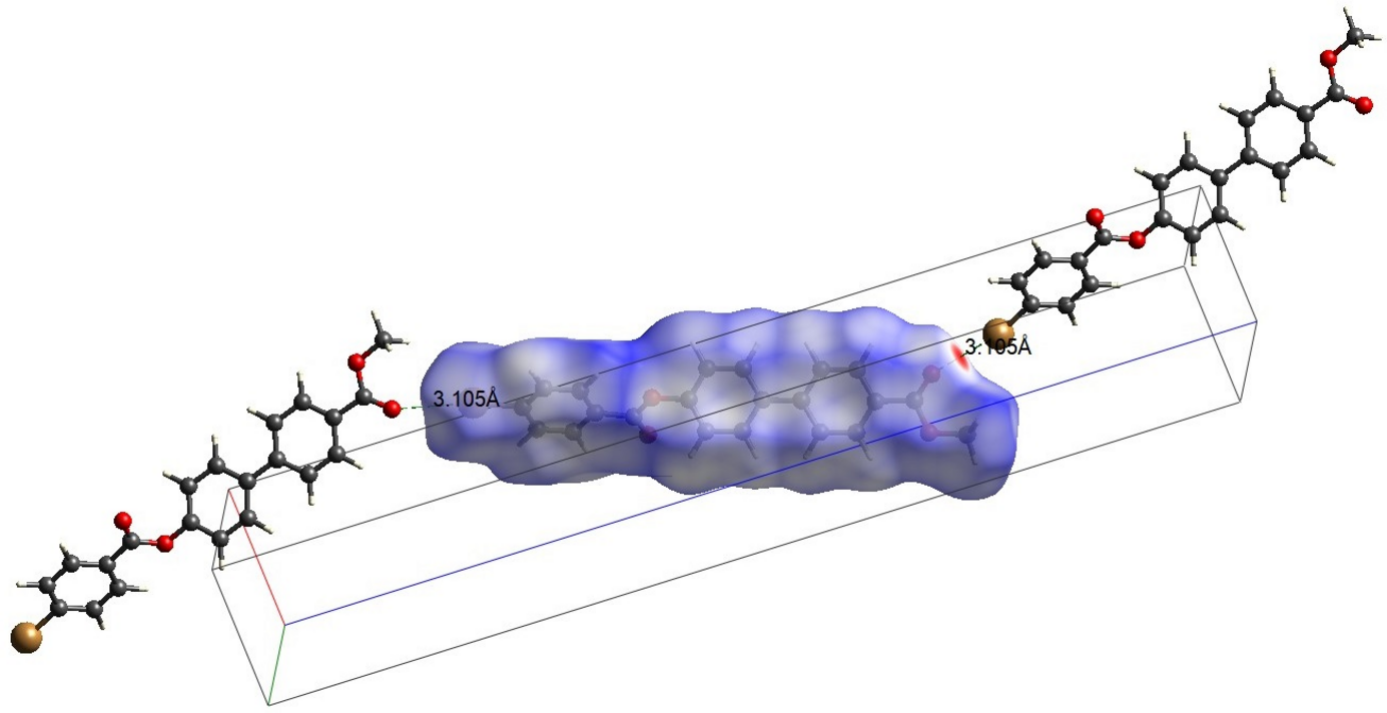
Hirshfeld surface of (I) mapped with *d*_norm_. The dashed lines indicate Br⋯O inter­actions running parallel [001].

**Figure 6:**
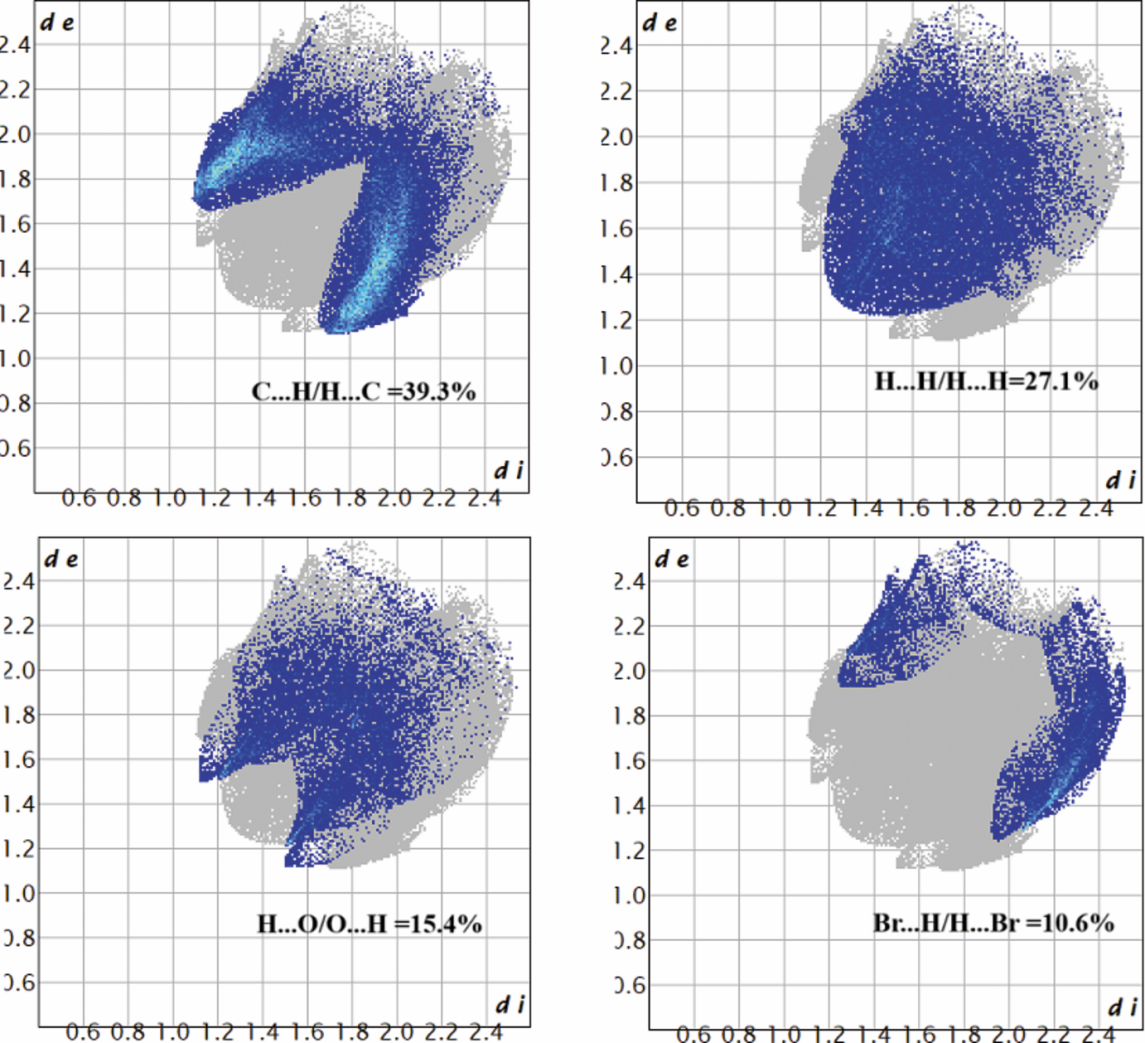
Two-dimensional fingerprint plots for (I).

Hirshfeld surface analysis was carried out using *CrystalExplorer* (Spackman *et al.*, 2021 ▸) to qu­antify the various inter­molecular inter­action present in (I). Fig. 4 ▸ illustrates the Hirshfeld surface mapped with *d*_norm_ where red spots near the oxygen atom of the surface correspond to the short contacts in the mol­ecule. The Br⋯O contact associated with electrophilic region of the Hirshfeld surface is shown in Fig. 5 ▸. The two-dimensional fingerprint plots (Fig. 6 ▸) reveal that the major contributions to the crystal packing are from H⋯H/H⋯H (27.1%), C⋯H/H⋯C (39.3%), O⋯H/H⋯O (15.4%) and Br⋯H/H⋯Br (10.6%) contacts.

**Figure 7:**
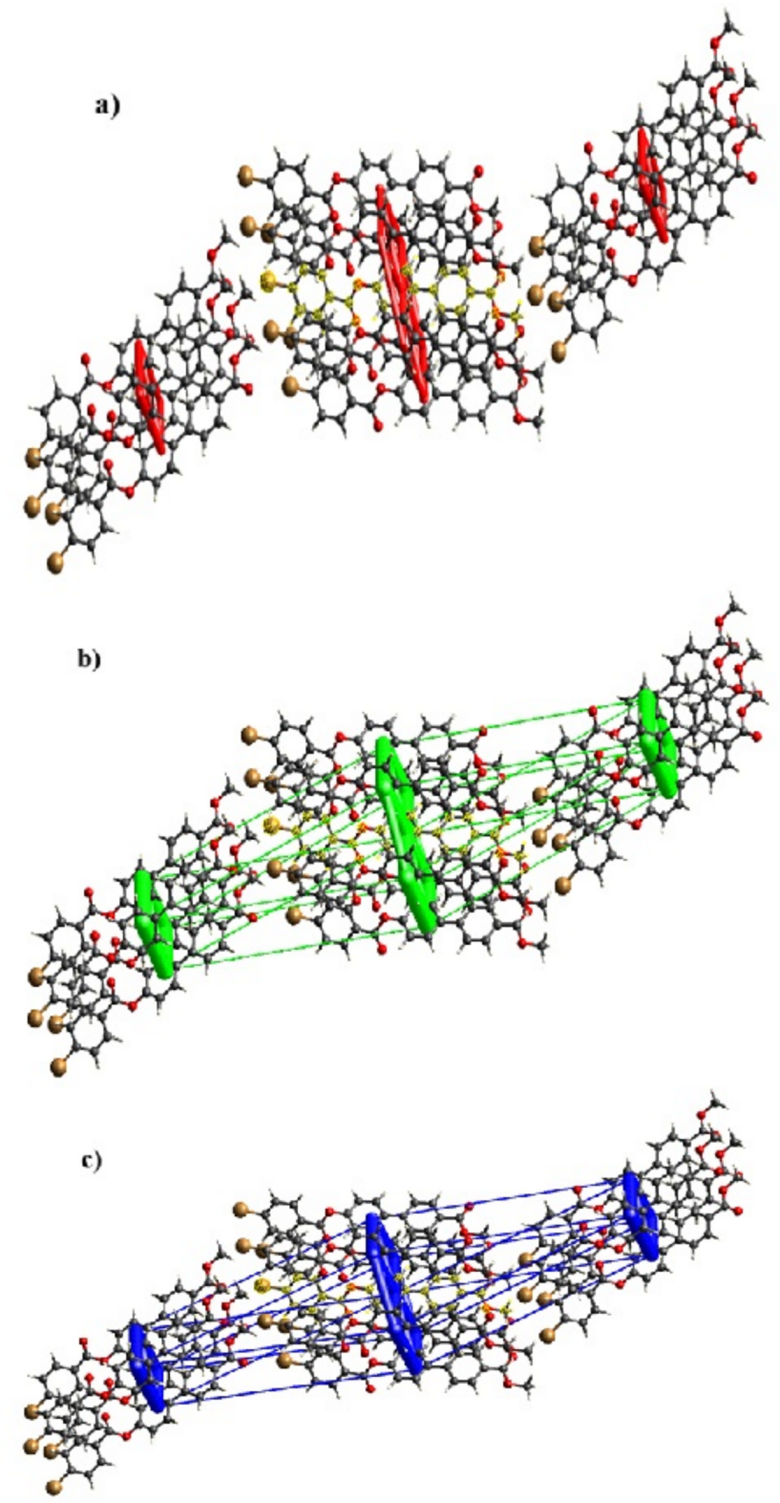
Energy frameworks calculated for (I), viewed along [100], showing (*a*) Coulomb potential force, (*b*) dispersion force and (*c*) total energy diagrams. The cylindrical radii are proportional to the relative strength of the corresponding energies (adjusted to a cutoff value of 5 kJ mol^−1^).

Energy framework calculations were performed using the basis set B3LYP /6-31G(d.p). The net inter­action energies for the title compound are *E*_ele_ = −41.9 kJ mol^−1^, *E*_pol_ = −11 kJ mol^−1^, *E*_dis_ = −209.7 kJ mol^−1^ and *E*_rep_ = 108.9 kJ mol^−1^, with a total inter­action energy *E*_tot_ of −167.9 kJ mol^−1^, which shows that *E*_dis_ is the major inter­action. The energy framework showing the electrostatic (coulomb) potential force, the dispersion force and the total energy diagram are shown in Fig. 7 ▸.

## 6. Density functional theory (DFT) calculations

**Figure 8:**
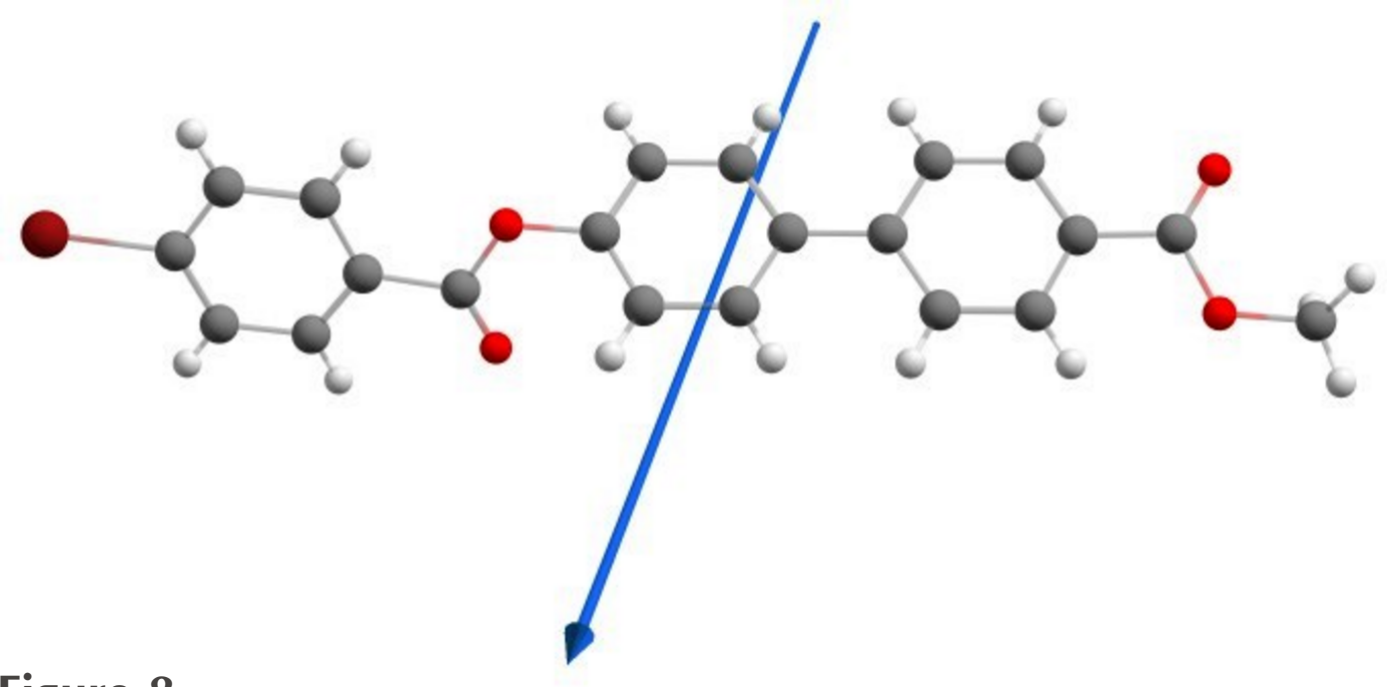
The direction of the dipole moment of (I) in the gaseous phase indicated by the arrow.

**Figure 9:**
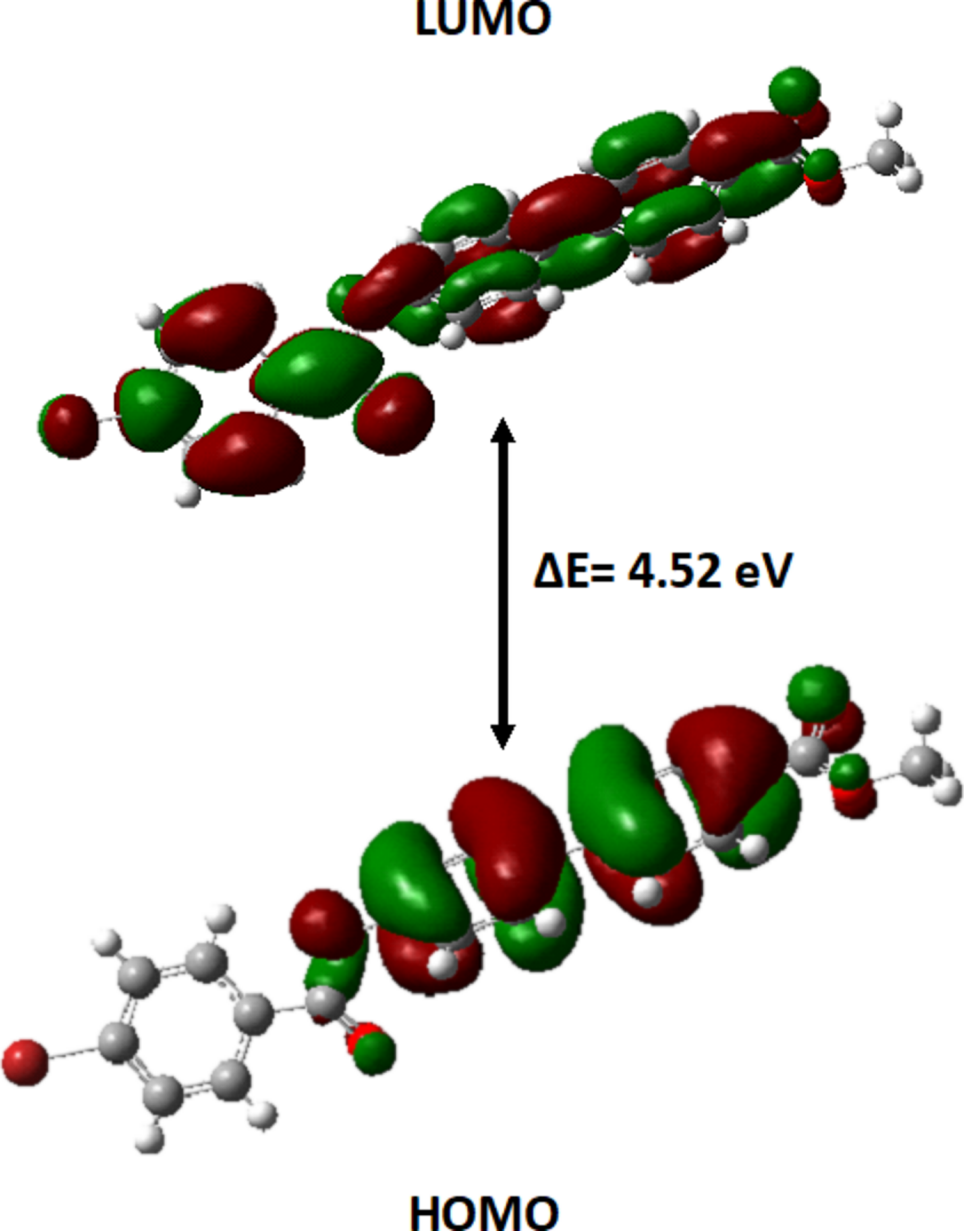
HOMO and LUMO of (I) with the energy band gap *E*_g_.

**Figure 10:**
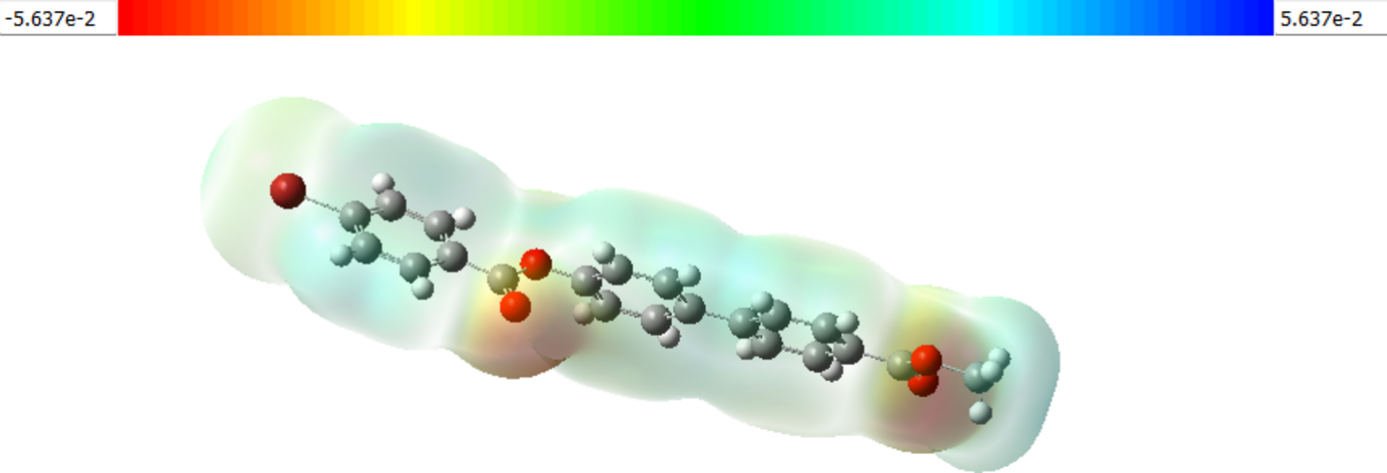
MEP surface plots of (I); regions of attractive potential appear in red and those of repulsive potential appear in blue.

DFT calculations were carried out using *Gaussian-09W* (Frisch *et al.*, 2009 ▸), with *Gaussian View 5.0* used to generate the optimized structure of (I). The optimized parameters of the title compound were obtained using the B3LYP/6-311G (d,p) basis set. The dipole moment of the mol­ecule in the gaseous phase was computed to be 1.2936 debye, and is illustrated in Fig. 8 ▸. Furthermore, the frontier mol­ecular orbitals HOMO and LUMO of (I) were computed, with energies of HOMO and LUMO of – 6.2450 and −1.7246 eV, respectively (the energy gap Δ*E* is 4.5203 eV; Fig. 9 ▸). The mol­ecular electrostatic potential (MEP) surface of the optimized structure is shown in Fig. 10 ▸. The nucleophilic and electrophilic reactive sites of (I) are represented by red and blue regions on the MEP surface. For (I), the red area covers the oxygen atoms of the ester functionality, revealing the sensitivity towards nucleophilic attack. The pale blue area around the aromatic rings indicates weak electrophilic sites.

## 7. Photophysical properties

**Figure 11:**
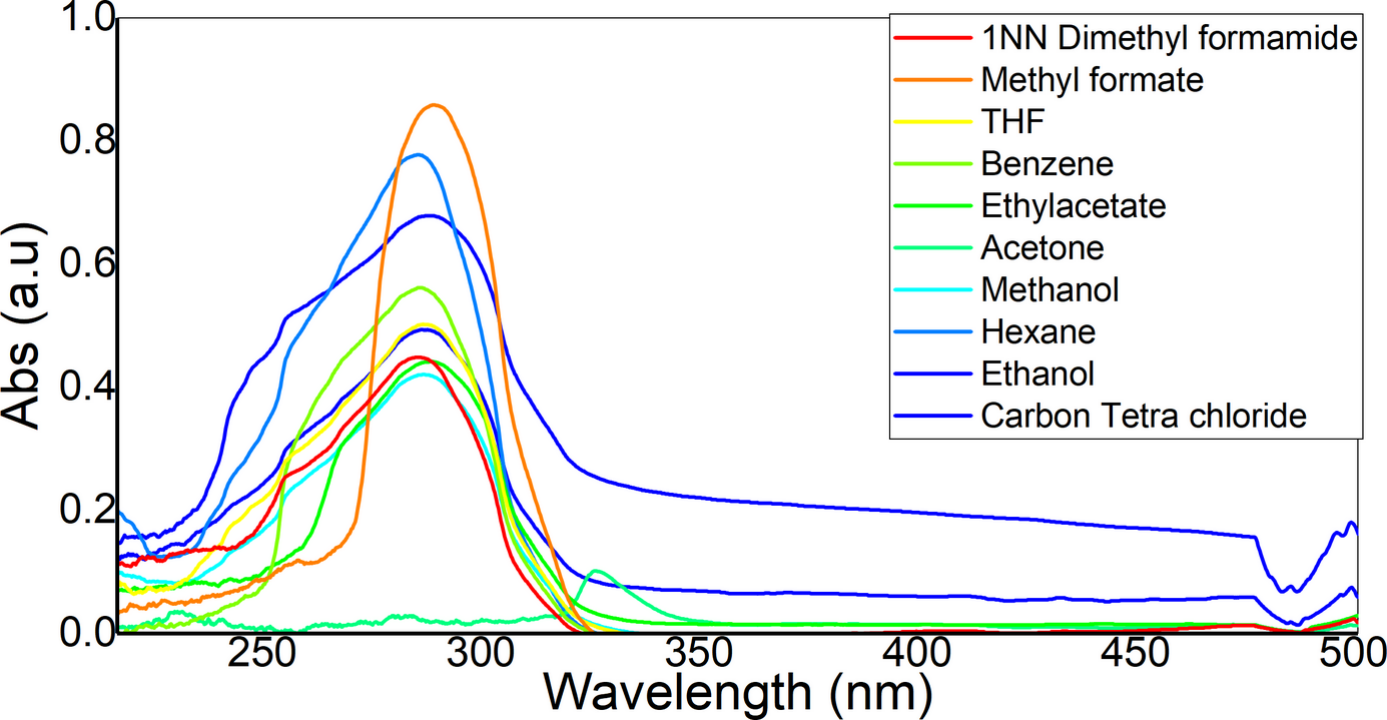
The absorption spectra of (I) recorded in different solvents.

**Figure 12:**
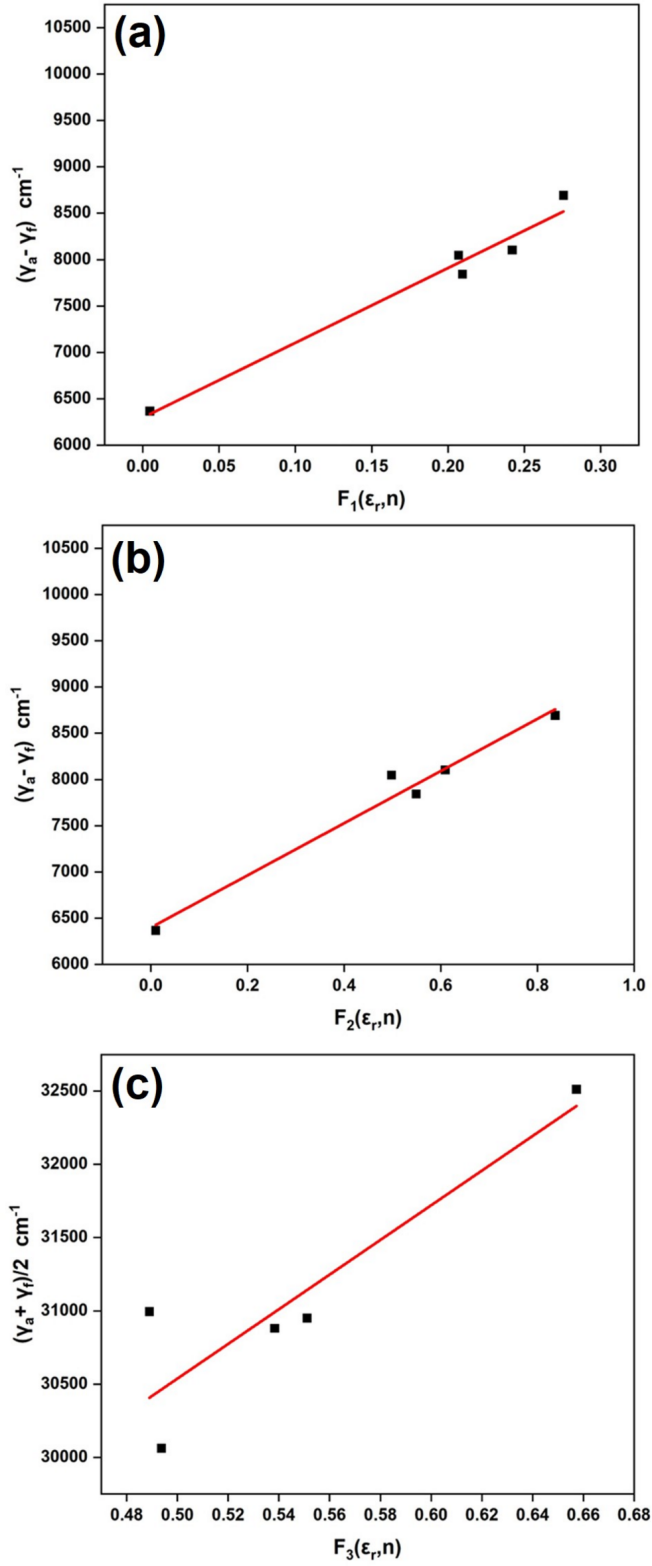
The variation of Stokes shift according to the (*a*) Lippert, (*b*) Bakshiev and (*c*) Bilot– Kawski–Chamma–Viallet functions in different solvents.

The photophysical properties of (I) were estimated by the solvatochromism method (Reichardt & Welton, 2011 ▸). The absorption spectra in different polar liquids were recorded, and intensity maxima observed between 285 and 288 nm (Fig. 11 ▸). Since the solvent polarity is susceptible to longer wavelength absorption, the samples were excited at longer wavelength to get emission spectra for calculation of the photophysical parameters. The ground state and experimental excited dipole moments were derived according to Lippert (1957 ▸), Bakhshiev (1964 ▸) and Bilot–Kawski–Chamma–Viallet (Bilot & Kawski, 1963 ▸; Chamma & Viallet, 1970 ▸) polarity functions, as detailed in equations (1)–(3). Among these, Bakhshiev and Bilot–Kawski–Chamma–Viallet equations give good results with reduced errors in the calculation. Fig. 12 ▸ shows 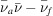
*versus*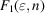, 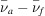
*versus*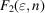 and (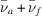)/2 *versus*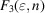, resulting in linear graphs with slopes *m_1_*, *m_2_* and *m_3_*, respectively.
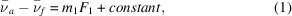

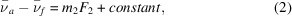

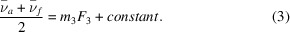
The expressions for the Lippert polarity [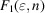] , Bakhshiev polarity [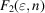] and Bilot–Kawski–Chamma–Viallet [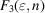] polarity functions are given by equations (4)– (6):
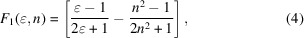

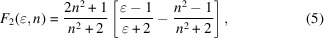

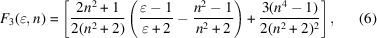
where 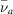 and 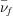 are the absorption and fluorescence maxima wavenumbers in cm^−1^, respectively; 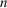 is the refractive index and 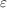 is the permittivity. The slopes 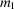, 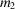, 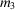 are connected with ground and exited state dipole moments through equations (7)– (9):
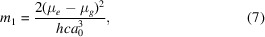

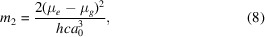

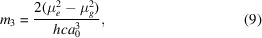
where μ_e_ and μ_g_ are the excited and ground state dipole moments of the solute mol­ecule (*h* and *c* are Planck’s constant and velocity of light in a vacuum, respectively); *a*_0_ is the Onsager cavity radius of the title compound as determined by Suppans’s equation *a_0_ =* (3*M*/4*πδN*)^*1/*3^ where *δ* is the density of the solute mol­ecule, *M* is mol­ecular weight and *N* is Avagadro’s number.
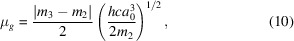

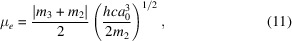

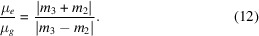
The solvent polarity function values 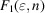, 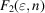, and 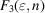 of various solvents on the band shift data of the title compound is summarized in Table 2 ▸. The slopes and inter­cepts of the fitted lines are given in Table 3 ▸ with good correlation coefficients obtained in all cases. The ground state and excited state dipole moments were estimated by the above equations under assumption that the symmetry of (I) remains unchanged upon electronic transition. The ground and excited dipole moments are found to be parallel according to equations (8) and (9). This part of the study demonstrates that the title compound is more polar in the excited state than in the ground state for all the solvents. The ratio of the dipole moments can be determined by Stokes shifts in different solvents as functions of ɛ and *n.* The calculated values for (I) are collated in Table 4 ▸.

## 8. Synthesis and crystallization

4-Bromo­benzoic acid (1 equiv.) was reacted with methyl 4′-hy­droxy-[1,1′-biphen­yl]-4-carboxyl­ate (1 equiv.) in dry chloro­form in the presence of di­cyclo­hexyl­carbodi­imide (1.2 equiv.) and a catalytic qu­antity of dimethyl amino­pyrimidine at room temperature for about 12 h. After completion of the reaction, the mixture was poured into water and extracted into chloro­form. The organic solvent was washed with water (10 ml), dilute acetic acid (10 ml) and dried over sodium sulfate. The crude final product was recrystallized from chloro­form at room temperature.

## 9. Refinement details

Crystal data, data collection and structure refinement details are summarized in Table 5 ▸. The structure was refined as a two-component inversion twin. All H atoms were positioned with idealized geometry and refined using a riding model with C—H = 0.93–0.96 Å and *U*_iso_(H) = 1.2–1.5*U*_eq_(C).

## Supplementary Material

Crystal structure: contains datablock(s) I. DOI: 10.1107/S2056989025001604/wm5751sup1.cif

Structure factors: contains datablock(s) I. DOI: 10.1107/S2056989025001604/wm5751Isup2.hkl

Supporting information file. DOI: 10.1107/S2056989025001604/wm5751Isup3.cml

CCDC reference: 2384236

Additional supporting information:  crystallographic information; 3D view; checkCIF report

## Figures and Tables

**Table 1:** Hydrogen-bond geometry (Å, °) *Cg*1, *Cg*2 and *Cg*3 are the centroids of the aromatic rings C1–C6, C8–C13 and C14–C19, respectively

*D*—H⋯*A*	*D*—H	H⋯*A*	*D*⋯*A*	*D*—H⋯*A*
C3—H3⋯*Cg*1^i^	0.93	2.82	3.526 (4)	133
C6—H6⋯*Cg*1^ii^	0.93	2.83	3.523 (4)	133
C10—H10⋯*Cg*2^iii^	0.93	2.83	3.523 (4)	132
C13—H13⋯*Cg*2^iv^	0.93	2.87	3.552 (4)	131
C16—H16⋯*Cg*3^iii^	0.93	2.92	3.566 (4)	128
C19—H19⋯*Cg*3^iv^	0.93	2.99	3.623 (4)	127

Symmetry codes: (i) 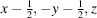; (ii) 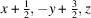; (iii) 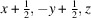; (iv) 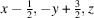.

**Table 2:** Solvatochromic data for (I) (cm^−1^) with calculated values of *F*_1_, *F*_2_ and *F*_3_

Solvent	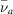	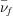	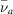 − 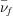	( 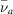 + 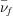 )/2	*F*_1_(ɛ, *n*)	*F*_2_(ɛ, *n*)	*F*_1_(ɛ, *n*)
Hexane	35018.66	25278.69	9739.96	30148.68	0.0010	0.0018	0.2540
Methyl formate	34931.50	26829.07	8102.43	30880.29	0.2421	0.6091	0.5383
Benzene	34580.37	28213.51	6366.85	31396.94	0.0045	0.0099	0.3430
THF	34872.48	27029.94	7842.53	30951.21	0.2095	0.5490	0.5511
CCl4	34665.78	25457.60	9208.18	30061.69	0.1434	0.3632	0.4936
Ethyl acetate	35018.66	26970.89	8047.76	30994.78	0.1996	0.4890	0.4979
Acetone	30659.54	28696.87	1962.67	29678.21	0.2841	0.7902	0.6395
Methanol	34872.48	30151.35	4721.12	32511.92	0.3083	0.8545	0.6514
Ethanol	34872.48	24628.11	10244.37	29750.30	0.2888	0.8129	0.6523
Dimethyl formamide	34665.782	25972.67	8693.10	30319.229	0.2757	0.8368	0.7077

**Table 3:** Statistical treatment of the correlations of solvent spectroscopic shifts of the title compound

Method	Slope	Inter­cept	Correlation coefficient	Number of data
Lippert correlation	8054	6928	0.97	5
Bakhshiev correlation	2820	6398	0.97	5
Bilot–Kawaski–Chamma–Viallet correlation	8626	24622	0.82	5

**Table 4:** Ground and excited states dipole moments of (I) (in debye^*a*^)

Mol­ecule	Radius *a* (Å)	μ_g_^*b*^ (D)	μ_g_^*c*^ (D)	μ_e_^*d*^ (D)	(μ_e_/μ_g_)^*e*^ (D)
(I)	4.6	1.2936	5.37	10.59	1.97

Notes: (*a*) 1 debye = 3.33564×10^−30^ cm = 10–18 esu cm; (*b*) the theoretical ground state dipole moment was obtained from *Gaussian 09*; (*c*) the experimental ground-state dipole moment was calculated according to equation (10); (*d*) the experimental excited dipole moment was calculated according to equation (11); (*e*) the ratio of excited state and ground state dipole moments was calculated using equation (12).

**Table 5:** Experimental details

Crystal data	
Chemical formula	C_21_H_15_BrO_4_
*M*_r_	411.24
Crystal system, space group	Orthorhombic, *Pna*2_1_
Temperature (K)	300
*a*, *b*, *c* (Å)	5.9754 (8), 7.2962 (9), 39.537 (5)
*V* (Å^3^)	1723.7 (4)
*Z*	4
Radiation type	Mo *K*α
μ (mm^−1^)	2.41
Crystal size (mm)	0.28 × 0.24 × 0.21

Data collection	
Diffractometer	Bruker *SMART* APEXII CCD
Absorption correction	Multi-scan (*SADABS*; Krause *et al.*, 2015 ▸)
*T*_min_, *T*_max_	0.152, 0.601
No. of measured, independent and observed [*I* > 2σ(*I*)] reflections	41401, 5204, 4189
*R*_int_	0.039
(sin θ/λ)_max_ (Å^−1^)	0.715

Refinement	
*R*[*F*^2^ > 2σ(*F*^2^)], *wR*(*F*^2^), *S*	0.035, 0.072, 1.05
No. of reflections	5204
No. of parameters	236
No. of restraints	1
H-atom treatment	H-atom parameters constrained
Δρ_max_, Δρ_min_ (e Å^−3^)	0.27, −0.46
Absolute structure	Refined as an inversion twin
Absolute structure parameter	0.060 (11)

Computer programs: *APEX2* and *SAINT* (Bruker, 2017 ▸), *SHELXT* (Sheldrick, 2015*a* ▸), *SHELXL* (Sheldrick, 2015*b* ▸), *Mercury* (Macrae *et al.*, 2020 ▸) and *publCIF* (Westrip, 2010 ▸).

## supplementary crystallographic information

### Methyl 4'-[(4-bromobenzoyl)oxy]biphenyl-4-carboxylate Crystal data

**Table:**

C_21_H_15_BrO_4_	*D*_x_ = 1.585 Mg m^−^^3^
*M**_r_* = 411.24	Melting point: 460 K
Orthorhombic, *P**n**a*2_1_	Mo *K*α radiation, λ = 0.71073 Å
Hall symbol: P 2c -2n	Cell parameters from 4189 reflections
*a* = 5.9754 (8) Å	θ = 2.8–30.0°
*b* = 7.2962 (9) Å	µ = 2.41 mm^−^^1^
*c* = 39.537 (5) Å	*T* = 300 K
*V* = 1723.7 (4) Å^3^	Prism, colourless
*Z* = 4	0.28 × 0.24 × 0.21 mm
*F*(000) = 832	

### Methyl 4'-[(4-bromobenzoyl)oxy]biphenyl-4-carboxylate Data collection

**Table:**

Bruker SMART APEXII CCD diffractometer	5204 independent reflections
Radiation source: fine-focus sealed tube	4189 reflections with *I* > 2σ(*I*)
Graphite monochromator	*R*_int_ = 0.039
Detector resolution: 1.02 pixels mm^-1^	θ_max_ = 30.6°, θ_min_ = 2.8°
φ and Ω scans	*h* = −8→8
Absorption correction: multi-scan (SADABS; Krause *et al.*, 2015)	*k* = −10→10
*T*_min_ = 0.152, *T*_max_ = 0.601	*l* = −55→56
41401 measured reflections	

### Methyl 4'-[(4-bromobenzoyl)oxy]biphenyl-4-carboxylate Refinement

**Table:**

Refinement on *F*^2^	Secondary atom site location: difference Fourier map
Least-squares matrix: full	Hydrogen site location: inferred from neighbouring sites
*R*[*F*^2^ > 2σ(*F*^2^)] = 0.035	H-atom parameters constrained
*wR*(*F*^2^) = 0.072	*w* = 1/[σ^2^(*F*_o_^2^) + (0.0182*P*)^2^ + 0.6795*P*] where *P* = (*F*_o_^2^ + 2*F*_c_^2^)/3
*S* = 1.05	(Δ/σ)_max_ < 0.001
5204 reflections	Δρ_max_ = 0.27 e Å^−^^3^
236 parameters	Δρ_min_ = −0.46 e Å^−^^3^
1 restraint	Absolute structure: Refined as an inversion twin
0.12 constraints	Absolute structure parameter: 0.060 (11)
Primary atom site location: structure-invariant direct methods	

### Methyl 4'-[(4-bromobenzoyl)oxy]biphenyl-4-carboxylate Special details

**Table:**

Geometry. All esds (except the esd in the dihedral angle between two l.s. planes) are estimated using the full covariance matrix. The cell esds are taken into account individually in the estimation of esds in distances, angles and torsion angles; correlations between esds in cell parameters are only used when they are defined by crystal symmetry. An approximate (isotropic) treatment of cell esds is used for estimating esds involving l.s. planes.
Refinement. Refined as a 2-component inversion twin.

### Methyl 4'-[(4-bromobenzoyl)oxy]biphenyl-4-carboxylate Fractional atomic coordinates and isotropic or equivalent isotropic displacement parameters (Å^2^)

**Table:**

	*x*	*y*	*z*	*U*_iso_*/*U*_eq_	
Br1	1.33516 (7)	0.54905 (5)	0.28015 (2)	0.05786 (12)	
O2	0.9870 (4)	0.5141 (3)	0.44334 (6)	0.0385 (5)	
O1	0.6870 (4)	0.3799 (4)	0.41979 (7)	0.0572 (7)	
C21	0.1186 (9)	0.5432 (8)	0.71793 (12)	0.0705 (15)	
H21A	−0.031918	0.589171	0.717313	0.106*	
H21B	0.117386	0.417942	0.725346	0.106*	
H21C	0.205866	0.615516	0.733346	0.106*	
O4	0.2160 (5)	0.5539 (4)	0.68434 (7)	0.0606 (8)	
C20	0.4255 (7)	0.4952 (5)	0.68076 (10)	0.0447 (8)	
C17	0.5027 (6)	0.5032 (5)	0.64515 (8)	0.0364 (7)	
C18	0.7117 (6)	0.4322 (5)	0.63731 (9)	0.0414 (8)	
H18	0.801659	0.385070	0.654425	0.050*	
C19	0.7869 (5)	0.4308 (5)	0.60442 (9)	0.0390 (7)	
H19	0.927359	0.382180	0.599737	0.047*	
C14	0.6585 (5)	0.5002 (4)	0.57790 (8)	0.0304 (6)	
C11	0.7408 (5)	0.5000 (4)	0.54250 (8)	0.0296 (6)	
C12	0.9405 (6)	0.4156 (5)	0.53322 (8)	0.0375 (7)	
H12	1.024510	0.356560	0.549743	0.045*	
C13	1.0183 (6)	0.4166 (5)	0.50024 (8)	0.0384 (7)	
H13	1.152969	0.359944	0.494794	0.046*	
C8	0.8942 (5)	0.5024 (4)	0.47567 (8)	0.0327 (6)	
C7	0.8675 (6)	0.4483 (4)	0.41677 (8)	0.0355 (7)	
C1	0.9865 (6)	0.4774 (4)	0.38438 (8)	0.0327 (6)	
C6	0.8794 (6)	0.4209 (5)	0.35476 (9)	0.0370 (8)	
H6	0.738063	0.367892	0.355958	0.044*	
C5	0.9820 (6)	0.4433 (5)	0.32362 (8)	0.0424 (8)	
H5	0.910596	0.406027	0.303881	0.051*	
C4	1.1928 (6)	0.5221 (5)	0.32236 (8)	0.0394 (7)	
C10	0.6189 (6)	0.5857 (5)	0.51662 (8)	0.0384 (7)	
H10	0.483925	0.642600	0.521791	0.046*	
C9	0.6957 (6)	0.5876 (5)	0.48333 (9)	0.0400 (8)	
H9	0.613348	0.645774	0.466508	0.048*	
C16	0.3731 (5)	0.5741 (5)	0.61941 (9)	0.0370 (7)	
H16	0.233589	0.623998	0.624316	0.044*	
C15	0.4492 (5)	0.5716 (5)	0.58628 (8)	0.0368 (7)	
H15	0.358554	0.618626	0.569238	0.044*	
C2	1.1985 (5)	0.5553 (4)	0.38240 (8)	0.0365 (7)	
H2	1.272007	0.591248	0.402036	0.044*	
C3	1.3003 (6)	0.5793 (5)	0.35129 (9)	0.0389 (7)	
H3	1.440710	0.633865	0.349921	0.047*	
O3	0.5380 (5)	0.4447 (5)	0.70399 (7)	0.0732 (10)	

### Methyl 4'-[(4-bromobenzoyl)oxy]biphenyl-4-carboxylate Atomic displacement parameters (Å^2^)

**Table:**

	*U*^11^	*U*^22^	*U*^33^	*U*^12^	*U*^13^	*U*^23^
Br1	0.0637 (2)	0.0753 (2)	0.03460 (15)	−0.00193 (19)	0.0122 (2)	0.0024 (2)
O2	0.0385 (12)	0.0495 (13)	0.0275 (10)	−0.0062 (10)	0.0031 (9)	−0.0048 (9)
O1	0.0510 (15)	0.0747 (18)	0.0459 (15)	−0.0271 (14)	0.0108 (12)	−0.0129 (14)
C21	0.069 (3)	0.101 (4)	0.041 (3)	0.004 (3)	0.015 (2)	0.003 (2)
O4	0.0532 (16)	0.092 (2)	0.0369 (15)	0.0124 (15)	0.0089 (12)	0.0039 (14)
C20	0.050 (2)	0.052 (2)	0.0323 (16)	−0.0042 (16)	0.0003 (15)	−0.0007 (14)
C17	0.0399 (16)	0.0391 (17)	0.0302 (14)	−0.0033 (13)	−0.0020 (13)	−0.0003 (13)
C18	0.0397 (17)	0.050 (2)	0.0351 (16)	0.0046 (15)	−0.0046 (13)	0.0008 (15)
C19	0.0328 (16)	0.050 (2)	0.0348 (16)	0.0074 (14)	−0.0039 (12)	0.0010 (14)
C14	0.0328 (13)	0.0256 (13)	0.0329 (15)	−0.0002 (12)	−0.0010 (12)	−0.0009 (11)
C11	0.0330 (14)	0.0247 (12)	0.0311 (14)	−0.0015 (11)	−0.0005 (12)	−0.0010 (11)
C12	0.0387 (16)	0.0393 (18)	0.0344 (16)	0.0076 (14)	−0.0010 (13)	0.0016 (13)
C13	0.0341 (16)	0.0447 (18)	0.0365 (17)	0.0087 (14)	0.0033 (13)	−0.0019 (13)
C8	0.0333 (15)	0.0322 (15)	0.0327 (15)	−0.0050 (12)	0.0044 (12)	−0.0031 (12)
C7	0.0390 (17)	0.0326 (15)	0.0350 (16)	−0.0014 (13)	0.0023 (13)	−0.0030 (13)
C1	0.0381 (15)	0.0279 (14)	0.0322 (15)	0.0001 (12)	−0.0005 (12)	−0.0015 (12)
C6	0.0348 (16)	0.0366 (16)	0.039 (2)	−0.0028 (12)	−0.0020 (14)	−0.0031 (14)
C5	0.0446 (18)	0.0495 (19)	0.0329 (17)	−0.0003 (15)	−0.0052 (14)	−0.0044 (14)
C4	0.0452 (19)	0.0421 (17)	0.0308 (16)	0.0024 (14)	0.0037 (13)	0.0025 (13)
C10	0.0381 (16)	0.0432 (18)	0.0339 (16)	0.0130 (14)	0.0028 (13)	0.0005 (13)
C9	0.0434 (19)	0.0443 (19)	0.0323 (15)	0.0120 (15)	−0.0019 (13)	0.0055 (13)
C16	0.0296 (16)	0.0448 (19)	0.0367 (16)	0.0039 (13)	0.0019 (12)	0.0009 (14)
C15	0.0353 (15)	0.0430 (18)	0.0322 (15)	0.0051 (14)	−0.0030 (12)	0.0058 (13)
C2	0.0357 (16)	0.0411 (17)	0.0327 (15)	−0.0014 (13)	−0.0026 (12)	−0.0019 (13)
C3	0.0340 (16)	0.0447 (18)	0.0379 (17)	−0.0030 (13)	0.0047 (13)	0.0006 (14)
O3	0.0623 (19)	0.125 (3)	0.0323 (14)	0.0227 (19)	−0.0003 (13)	0.0077 (16)

### Methyl 4'-[(4-bromobenzoyl)oxy]biphenyl-4-carboxylate Geometric parameters (Å, º)

**Table:**

Br1—C4	1.884 (3)	C12—C13	1.384 (4)
O2—O2	0.000 (4)	C12—H12	0.9300
O2—C7	1.358 (4)	C13—C8	1.373 (5)
O2—C8	1.396 (4)	C13—H13	0.9300
O1—C7	1.195 (4)	C8—C9	1.373 (5)
C21—O4	1.452 (5)	C7—C1	1.480 (4)
C21—H21A	0.9600	C1—C2	1.390 (4)
C21—H21B	0.9600	C1—C6	1.397 (5)
C21—H21C	0.9600	C6—C5	1.385 (5)
O4—C20	1.331 (5)	C6—H6	0.9300
C20—O3	1.196 (5)	C5—C4	1.386 (5)
C20—C17	1.483 (5)	C5—H5	0.9300
C17—C16	1.379 (5)	C4—C3	1.376 (5)
C17—C18	1.387 (5)	C10—C9	1.394 (5)
C18—C19	1.376 (5)	C10—H10	0.9300
C18—H18	0.9300	C9—H9	0.9300
C19—C14	1.394 (4)	C16—C15	1.387 (4)
C19—H19	0.9300	C16—H16	0.9300
C14—C15	1.395 (4)	C15—H15	0.9300
C14—C11	1.484 (4)	C2—C3	1.384 (5)
C11—C12	1.392 (5)	C2—H2	0.9300
C11—C10	1.403 (4)	C3—H3	0.9300
			
C7—O2—C8	118.6 (3)	C9—C8—O2	121.2 (3)
O4—C21—H21A	109.5	C13—C8—O2	117.4 (3)
O4—C21—H21B	109.5	O1—C7—O2	123.0 (3)
H21A—C21—H21B	109.5	O1—C7—C1	125.5 (3)
O4—C21—H21C	109.5	O2—C7—C1	111.5 (3)
H21A—C21—H21C	109.5	C2—C1—C6	119.4 (3)
H21B—C21—H21C	109.5	C2—C1—C7	123.0 (3)
C20—O4—C21	117.2 (3)	C6—C1—C7	117.6 (3)
O3—C20—O4	123.1 (4)	C5—C6—C1	120.5 (3)
O3—C20—C17	124.5 (4)	C5—C6—H6	119.7
O4—C20—C17	112.4 (3)	C1—C6—H6	119.7
C16—C17—C18	118.7 (3)	C6—C5—C4	118.9 (3)
C16—C17—C20	122.7 (3)	C6—C5—H5	120.6
C18—C17—C20	118.5 (3)	C4—C5—H5	120.6
C19—C18—C17	120.5 (3)	C3—C4—C5	121.4 (3)
C19—C18—H18	119.7	C3—C4—Br1	119.6 (3)
C17—C18—H18	119.7	C5—C4—Br1	119.1 (3)
C18—C19—C14	121.9 (3)	C9—C10—C11	121.5 (3)
C18—C19—H19	119.0	C9—C10—H10	119.3
C14—C19—H19	119.1	C11—C10—H10	119.3
C19—C14—C15	116.8 (3)	C8—C9—C10	119.2 (3)
C19—C14—C11	121.8 (3)	C8—C9—H9	120.4
C15—C14—C11	121.4 (3)	C10—C9—H9	120.4
C12—C11—C10	116.7 (3)	C17—C16—C15	120.5 (3)
C12—C11—C14	122.2 (3)	C17—C16—H16	119.7
C10—C11—C14	121.1 (3)	C15—C16—H16	119.7
C13—C12—C11	122.3 (3)	C16—C15—C14	121.6 (3)
C13—C12—H12	118.9	C16—C15—H15	119.2
C11—C12—H12	118.9	C14—C15—H15	119.2
C8—C13—C12	119.2 (3)	C3—C2—C1	120.2 (3)
C8—C13—H13	120.4	C3—C2—H2	119.9
C12—C13—H13	120.4	C1—C2—H2	119.9
C9—C8—C13	121.1 (3)	C4—C3—C2	119.7 (3)
C9—C8—O2	121.2 (3)	C4—C3—H3	120.2
C13—C8—O2	117.4 (3)	C2—C3—H3	120.2
			
C21—O4—C20—O3	−4.2 (6)	O2—C7—C1—C2	2.7 (4)
C21—O4—C20—C17	176.9 (4)	O1—C7—C1—C6	0.2 (5)
O3—C20—C17—C16	−175.4 (4)	O2—C7—C1—C6	−178.2 (3)
O4—C20—C17—C16	3.5 (5)	O2—C7—C1—C6	−178.2 (3)
O3—C20—C17—C18	6.2 (6)	C2—C1—C6—C5	−0.5 (5)
O4—C20—C17—C18	−174.8 (3)	C7—C1—C6—C5	−179.7 (3)
C16—C17—C18—C19	−0.7 (5)	C1—C6—C5—C4	0.2 (5)
C20—C17—C18—C19	177.7 (3)	C6—C5—C4—C3	−0.5 (5)
C17—C18—C19—C14	0.2 (5)	C6—C5—C4—Br1	178.9 (3)
C18—C19—C14—C11	179.5 (3)	C12—C11—C10—C9	−0.6 (5)
C19—C14—C11—C12	6.1 (5)	C14—C11—C10—C9	179.5 (3)
C15—C14—C11—C12	−174.4 (3)	C13—C8—C9—C10	−0.3 (5)
C19—C14—C11—C10	−174.0 (3)	O2—C8—C9—C10	−174.5 (3)
C15—C14—C11—C10	5.5 (5)	O2—C8—C9—C10	−174.5 (3)
C10—C11—C12—C13	0.6 (5)	C11—C10—C9—C8	0.5 (5)
C14—C11—C12—C13	−179.5 (3)	C18—C17—C16—C15	1.0 (5)
C11—C12—C13—C8	−0.5 (5)	C20—C17—C16—C15	−177.3 (3)
C12—C13—C8—C9	0.3 (5)	C17—C16—C15—C14	−0.8 (5)
C12—C13—C8—O2	174.7 (3)	C19—C14—C15—C16	0.3 (5)
C12—C13—C8—O2	174.7 (3)	C11—C14—C15—C16	−179.2 (3)
C7—O2—C8—C9	−60.4 (4)	C6—C1—C2—C3	1.1 (5)
C7—O2—C8—C13	125.3 (3)	C7—C1—C2—C3	−179.8 (3)
C8—O2—C7—O1	−0.5 (5)	C5—C4—C3—C2	1.1 (5)
C8—O2—C7—C1	178.0 (3)	Br1—C4—C3—C2	−178.3 (3)
O1—C7—C1—C2	−178.9 (3)	C1—C2—C3—C4	−1.4 (5)
O2—C7—C1—C2	2.7 (4)		

### Methyl 4'-[(4-bromobenzoyl)oxy]biphenyl-4-carboxylate Hydrogen-bond geometry (Å, º)

*Cg*1, *Cg*2 and *Cg*3 are the centroids of the aromatic rings C1–C6, C8–C13 and C14–C19, respectively

**Table:**

*D*—H···*A*	*D*—H	H···*A*	*D*···*A*	*D*—H···*A*
C3—H3···*Cg*1^i^	0.93	2.82	3.526 (4)	133
C6—H6···*Cg*1^ii^	0.93	2.83	3.523 (4)	133
C10—H10···*Cg*2^iii^	0.93	2.83	3.523 (4)	132
C13—H13···*Cg*2^iv^	0.93	2.87	3.552 (4)	131
C16—H16···*Cg*3^iii^	0.93	2.92	3.566 (4)	128
C19—H19···*Cg*3^iv^	0.93	2.99	3.623 (4)	127

Symmetry codes: (i) *x*−1/2, −*y*−1/2, *z*; (ii) *x*+1/2, −*y*+3/2, *z*; (iii) *x*+1/2, −*y*+1/2, *z*; (iv) *x*−1/2, −*y*+3/2, *z*.
